# The Liver Knows: Preoperative MELD Score as a Predictor of Outcome in Patients Undergoing Left Ventricular Assist Device Implantation. A Single‐Center Retrospective Cohort Study

**DOI:** 10.1002/hsr2.71071

**Published:** 2025-07-23

**Authors:** Mohamed Elbayomi, Presheet Pathare, Raphael Groß, Friedrich Mellert, Oliver Dewald

**Affiliations:** ^1^ Department of Cardiac Surgery Friedrich‐Alexander‐University Erlangen Bavaria Germany

**Keywords:** left ventricle assist device, liver function, risk stratification

## Abstract

**Background and Aims:**

Right‐side heart failure (RSHF) jeopardizes left ventricular assist device (LVAD) short‐ and long‐term outcomes. The model for end‐stage liver disease (MELD) score is an effective means of evaluating liver dysfunction. This study aims to investigate the predictive utility of preoperative MELD on post‐LVAD implantation outcomes, specifically focusing on the incidence of RSHF.

**Methods:**

This single‐center retrospective cohort study included 133 patients who received durable continuous‐flow LVADs with a centrifugal pump from 2015 to 2022. The primary outcome was RSHF, defined as necessitating right ventricular (RV), temporary or durable, mechanical support. The research hypothesis was that a high preoperative MELD score is associated with a higher incidence of RSHF after LVAD implantation.

**Results:**

The overall post‐LVAD RSHF incidence was 18% (*n* = 24), and 90‐day mortality was 30% (*n* = 40). The mean MELD score was 14.7 (±6.9). RSHF was significantly associated with in‐hospital mortality (Peasrson's chi‐squared = 37.86, *p* < 0.001). The RSHF group had a higher mean MELD score of 18.7 (±2), whereas the control group had a mean MELD score of 13.8 (±0.5). The mean MELD difference between the RSHF and non‐RSHF groups was 4.75 (95% CI: 1.7–7.7), with the RSHF group having a higher mean and (18.7 ± 2, *p* = 0.001) compared to the control group (14 ± 0.5, *p* = 0.002). The incidence of RSHF did not statistically differ between INTERMACS groups (*p* = 0.35). Preoperative MELD score was an independent predictor of RSHF in a multivariable logistic regression model, including age, EuroSCORE‐2, and INTERMACS categories (OR 1.08; 95% CI: 1.02–1.15; *p* = 0.009).

**Conclusion:**

Preoperative MELD score may predict the incidence of postoperative RSHF in LVAD patients. Candidates with elevated MELD scores should be carefully evaluated for alternative therapeutic modalities or optimized aggressively before LVAD implantation.

## 1 Introduction

Durable mechanical circulatory support devices, such as left ventricular assist devices (LVADs), have improved heart failure outcomes compared to medical treatment alone [1]. Right ventricle (RV) failure after left ventricle assist device implantation is a developing complication that has been associated with increased mortality [2]. Restoring cardiac function and reducing mortality is the ultimate objective of heart failure treatment [3].

The Model for End‐Stage Liver Disease (MELD) score is a simple, reliable predictor of outcomes, especially in high‐risk patients undergoing heart surgery with cardiopulmonary bypass. Preoperative risk stratification of the development of right‐sided heart failure (RSHF) in patients who receive an LVAD is crucial. End‐stage liver failure in patients undergoing LVAD implantation is often associated with an increased mortality because it can result in multiple organ failure [4].

Evaluation of patients undergoing LVAD implantation for the risk of developing RSHF is performed using different appropriate scores, for example, EUROMACS‐RHF, Seattle Heart Failure model, etc., which considers various hemodynamic parameters to assess the RV function. Risk stratification helps healthcare providers better select patients, be prepared for possible complications, improve results, and reduce costs. This single‐centre retrospective study aims to identify the correlation between the MELD score and the incidence of severe RSHF after LVAD implantation.

## 2 Patients and Methods

### 2.1 Study Design and Patient Population

The study hypothesized that a higher MELD score is associated with a higher incidence of severe acute and subacute RSHF after LVAD implantation.

**Figure 1:**
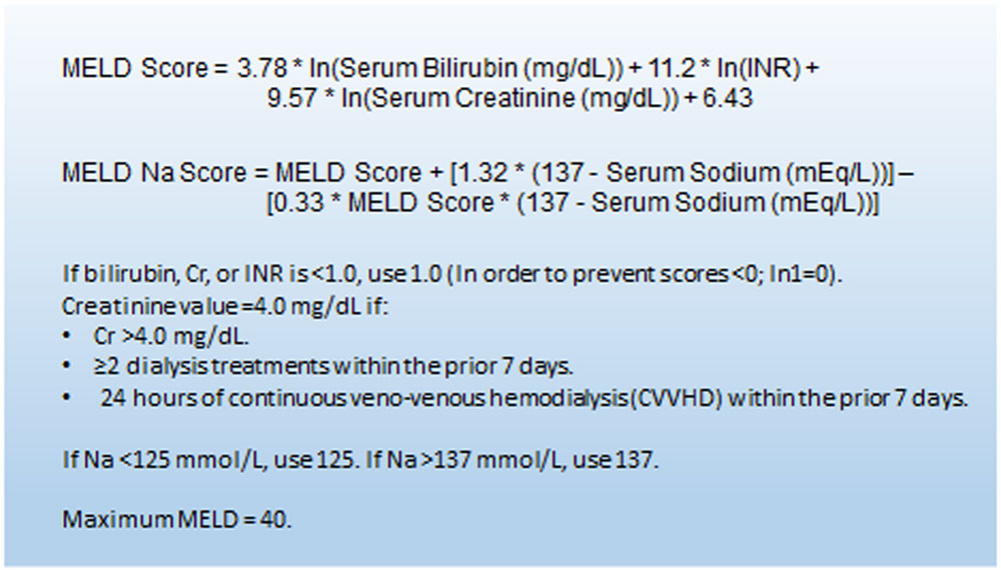
MELD score calculation.

We undertook a single‐center retrospective cohort study using our cardiothoracic database and electronic patient records at an acute care university hospital in northern Bavaria, Germany, from the year 2000 through 2022, were analyzed for adult patients undergoing LVAD implantation. Four hundred and forty‐six patients were included in the initial analysis and they all had a documented preoperative bilirubin, creatinine, or INR to measure every patient's MELD score. The MELD score of the patients was calculated using the formula shown in Figure 1.

Patients were then excluded based on the following criteria.

- Preoperative implantation of ECLS/ECMO.
- Implantation of an assist device as an emergency/salvage procedure.
- Patients receiving concomitant procedures (e.g., valve replacement/repair, CABG).
- Implantation of extracorporeal LVADs.

The MELD score was calculated, and the patients were then classified into four groups based on their MELD score: MELD < 10 (Group 1), MELD 11–20 (Group 2), MELD 21–30 (Group 3), and MELD > 30 (Group 4) [5].

Finally, 133 adults (> 18 years of age), who underwent implantation of a continuous flow left ventricle assist device (LVAD) with a centrifugal pump, were included in the study.

The primary endpoint of this study was defined as severe acute and subacute RSHF after LVAD implantation, necessitating the implantation of a temporary or durable right ventricular assist device (RVAD). Consistent with contemporary definitions such as those proposed by the Mechanical Circulatory Support–Academic Research Consortium, our analysis focused on RSHF events occurring intraoperatively and within the initial 30 days following LVAD implantation. We used the implantation of ECMO/ECLS as a well‐defined criteria as this has the most impact on patient survival and is not open to interpretation when compared to other subjective/user‐derived measured criteria for RSHF.

Late‐stage RHF (occurring after 30 days postimplantation) was not assessed as a primary or secondary endpoint in this study. Secondary endpoint was established as in‐hospital mortality.

#### 2.1.1 Human Participant Protection

This retrospective study complied with the Helsinki Declaration (2000), and the local Ethics Committee (EC, No. 22‐448‐Br) approved this analysis based on retrospective data retrieval; the EC waived the need for written patients’ informed consent.

#### 2.1.2 Data Collection and Statistical Analysis

All patient‐associated data were collected in an approved manner on a comprehensive spreadsheet. Continuous variables are presented as mean ± SD, median and interquartile range (IQR), as appropriate. Categorical variables are presented as frequencies and percentages. Comparisons between groups were performed using Student's *t*‐test or one‐way ANOVA for continuous variables and the chi‐squared test for categorical variables, as appropriate. For non‐normally distributed continuous variables, the Wilcoxon rank‐sum test was used. A multivariable logistic regression model was constructed to identify independent predictors of RSHF and 90‐day mortality. The variables included in the multivariable models were chosen based on clinical relevance and univariable associations. All statistical tests were two‐sided, and a *p* < 0.05, was considered statistically significant. Statistical analyses were performed using Stata/SE statistical software version 17.0 (StataCorp., College Station, TX, USA). No specific a priori hypotheses were defined for subgroup analyses, which should therefore be considered exploratory.

## 3 Results

The mean age in this study was 55 (SD ± 11), 116 patients (87%) were males, and the mean body mass index (BMI) of the patients included was 26.8 (SD ± 4). The mean EuroSCORE II of the patients was 9 (SD ± 3.4). The MELD score of this cohort's patients was 14.8 (SD ± 6.9). The prevalence of diabetes mellitus in our cohort was 30% (*n* = 38). At the time of presentation, 32% (*n* = 42) of patients were in INTERMACS Class I (Table 1). The most common cause for left ventricular insufficiency was ischemic cardiomyopathy, with 45% (*n* = 60) and dilated cardiomyopathy was the second most common cause with 40% (*n* = 53) (Table 2). The median left ventricle ejection fraction was 15, with an IQR between 10 and 20. In 119 patients (89%), HVAD (HeartWare) was implanted as a left ventricle assist device, and the remaining patients received the HeartMate 3.

**Table 1:** Baseline patient characteristics.

Baseline characteristics^a^	
Age of patient, years	55 ± 10.8
Female sex. *n* (%)	16 (12)
Body mass index (BMI) (kg/m^2^)	26.8 ± 4.3
Diabetes mellitus, *n* (%)	38 (28.5)
EuroSCORE II	9.1 ± 3.4
MELD score	14.8 ± 6.9
MELD Na^+^ score	16.1 ± 7.6
Cardiogenic shock at presentation, *n* (%)	39 (29)
Etiology, *n* (%)	
Ischemic cardiomyopathy	63 (47)
Dilated cardiomyopathy	53 (40)
Non‐compaction cardiomyopathy	5 (3.7)
Myocarditis	5 (3.7)
Others	7 (5.6)
Left ventricle ejection fraction in echocardiography (%)	17 ± 18
Bypass time (minutes)	57 ± 24
HeartWare Medtronic LVAD, *n* (%)	119 (89.4)
INTERMACS Class I, *n* (%)	42 (31.5)
Severe Right‐sided heart failure (RSHF), *n* (%)	24 (18)
90‐day mortality, *n* (%)	41 (31)

^a^ Plus‐minus values are means ± SD. There were 133 patients in the study. The baseline score was collected preoperatively.

**Table 2:** Stratification of patients' preoperative characteristics and postoperative outcomes according to their preoperative NYHA Score.

Stratification of patients according to their preoperative MELD score					
MELD score	< 9.9 (*n* = 30)	10–19.9 (*n* = 75)	20–29.9 (*n* = 22)	> 29.9 (*n* = 6)	*p* value
Preoperative factors					
Age of patient, years	55 ± 10.5	56 ± 11	56 ± 9	51 ± 5.7	0.35
EuroSCORE II	8.4 ± 3.5	9.2 ± 3.3	9.7 ± 3.7	10.2 ± 2.4	0.79
BMI (kg/m^2^)	26 ± 4	26.7 ± 4	27 ± 5	30 ± 3	0.62
Preoperative serum creatinine (mg/dL)	0.9 ± 0.6	1.2 ± 0.75	1.7 ± 0.9	3.5 ± 1.8	< 0.001*
Preoperative serum bilirubin (mg/dL)	0.8 ± 0.3	1.6 ± 1.5	2.5 ± 1.7	7.9 ± 3.7	< 0.001*
MELD Na^+^	7.7 ± 1	15.6 ± 4.5	24.4 ± 3.2	37 ± 3.6	< 0.001*
Left ventricular ejection fraction in echocardiography	27 ± 39	17.5 ± 19	16 ± 18	16.2 ± 8	< 0.001*
Intra‐ and postoperative factors					
Duration of operation (min)	164.7 ± 40.	186 ± 63	171 ± 39	197 ± 21.5	0.002*
Severe RSHF, *n* (%)	5 (16.6)	11 (14.4)	4 (18)	4 (80)	0.003*
Re‐thoracotomy because of bleeding, *n* (%)	6 (20)	12 (16)	7 (31)	3 (60)	0.065
Postoperative intensive care unit (ICU), days	9.3 ± 11.9	14.4 ± 19	18 ± 19.9	20 ± 14.4	0.03*

*Note:* Plus‐minus values are means ± SD. One hundred and thirty‐three patients are included in the study and have been stratified based on their preoperative MELD score.

* Significant *p* value.

After categorizing the patients according to their MELD score with the ranges described in the Methods section, as shown in Table 3, there was a difference in the length of stay in the intensive care unit between the different MELD score categories, as Group 1 had the shortest length of stay in the ICU with a mean of 9 days, while Group 4 had the longest with a mean of 20 days. There was a difference between the different categories of MELD score and the re‐thoracotomy due to bleeding. However, the difference was marginal and did not reach a statistically significant chi‐squared score with 3 degrees of freedom (df) of 7.2, yielding a *p* value of 0.065.

**Table 3:** 90‐day mortality due to severe right‐sided heart failure after LVAD implantation.

Severe right‐sided heart failure after LVAD implantation	90‐days mortality		
	No	Yes	Total
No	88	21	109
	80.73%	19.27%	100.00%
	95.65%	51.22%	81.95%
Yes	4	20	24
	16.67%	83.33%	100.00%
	4.35%	48.78%	18.05%
Total	92	41	133
	69.17%	30.83%	100.00%
	100.00%	100.00%	100.00%

*Note:* Pearson *χ* ^2^ = 37.86, *p* < 0.0001.

The first row has frequencies; the second row has row percentages, and the third row has column percentages.

Other scores included in this study did not demonstrate a statistically significant association with the incidence of RSHF. A statistically significant difference in mean EuroSCORE II was not observed between the groups (*p* = 0.8). No statistically significant difference in RSHF incidence was found across INTERMACS classifications using the chi‐squared test of independence (*p* = 0.43).

**Figure 2:**
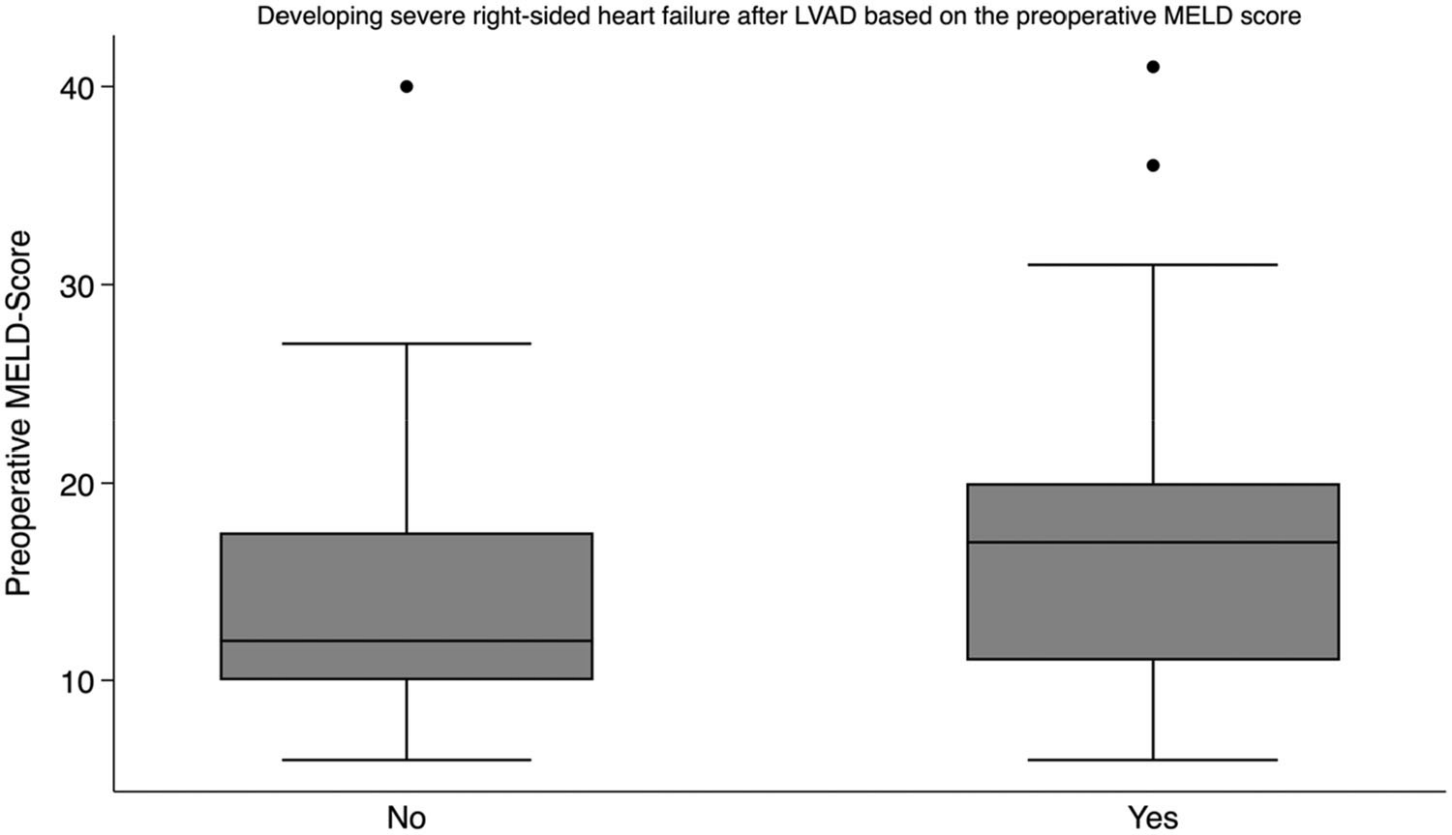
Development of right‐sided heart failure‐Box plot showing the median with the 25th and 75th percentile of the preoperative MELD score difference in the groups based on the development of severe right‐sided heart failure after left ventricle assist device implantation.

Severe RSHF (as defined in the Methods section) occurred in 18% (*n* = 24) of patients. Ninety‐day mortality was 30% (*n* = 40). Using a chi‐squared test of independence, the incidence of severe RSHF was significantly associated with 90‐day mortality with a Pearson chi‐squared score of 37.8 and *p* < 0.0001 (Table 3). Using a *t*‐test, the group that did not develop severe RSHF had a mean MELD score of 14 with 95% CI (12.89–15.1), while the group that developed severe RSHF had a mean MELD score of 18.75 with 95% CI (14.5–22.9). The difference in mean MELD score between the two groups was 4.75 (95% CI: 1.7–7.7; *p* = 0.002). Using the two‐sample Wilcoxon rank‐sum (Mann–Whitney) test, a statistically significant difference was observed in preoperative MELD score between the RSHF and non‐RSHF groups (*Z* = −2.05; *p*= 0.04). Graphical representation of each group's median and the 25th and 75th percentile is demonstrated in Figure 2.

We further analyzed the data of two patients who exhibited contrasting outcomes following LVAD implantation despite having MELD and MELD Na scores of 40, indicating an elevated risk of postoperative complications. As predicted, one patient developed right heart failure (RHF) post‐LVAD. Despite his high MELD score, the second patient defied expectations by remaining free of RHF and surviving to discharge.

A closer examination of this outlier case revealed specific preoperative factors potentially contributing to the favorable outcome. First, while the patient presented with elevated creatinine, his sodium levels remained within normal limits. This finding suggests the presence of partially compensated renal insufficiency, implying a potentially adaptive mechanism. Second, this patient received preoperative mechanical circulatory support in the form of an intra‐aortic balloon pump (IABP) support for 12 h. This intervention could have provided crucial diastolic support to the left ventricle, leading to a reduction in postcapillary pulmonary pressure (PCP).

In our examination, we found that about 31% of patients presented with INTERMACS 1. Despite the proportion of patients presenting with severe heart failure, we observed that only ca. 18% of patients developed severe RSHF. Instead, we found that this correlated with the 90‐day mortality of about 30%. This might mean that patients who presented with severe heart failure are at a higher risk of mortality, despite appropriate treatment.

**Figure 3:**
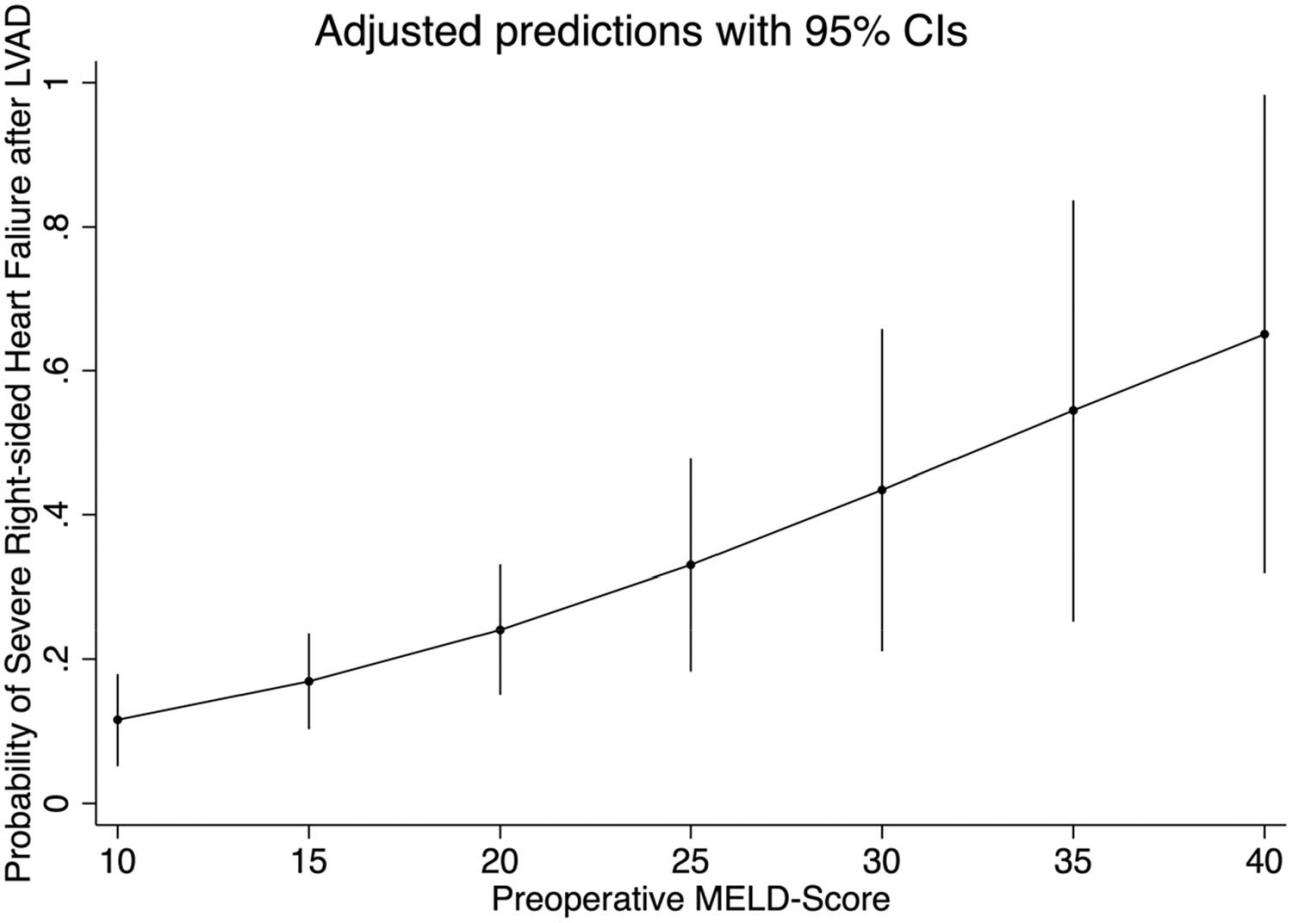
Predictive ability of the MELD score‐marginal plot demonstrating the relation between the preoperative MELD score and the probability of developing severe right‐sided heart failure after left ventricle assist device implantation, including the 95% confidence interval.

A multivariable logistic regression module included the MELD score, age of the patient at the LVAD implantation, EuroSCORE II, New York Heart Association Functional classification, and the etiology of myopathy, and showed MELD score as an independent predictor of severe RSHF (OR 1.08, 95% CI [1.0–1.1], *p* value 0.007), which can be interpreted as every one point increases in MELD score increases the probability of RSHF by 8%, we developed marginal plot to show the relation between the two variables with a 95% confidence interval (Figure 3).

**Figure 4:**
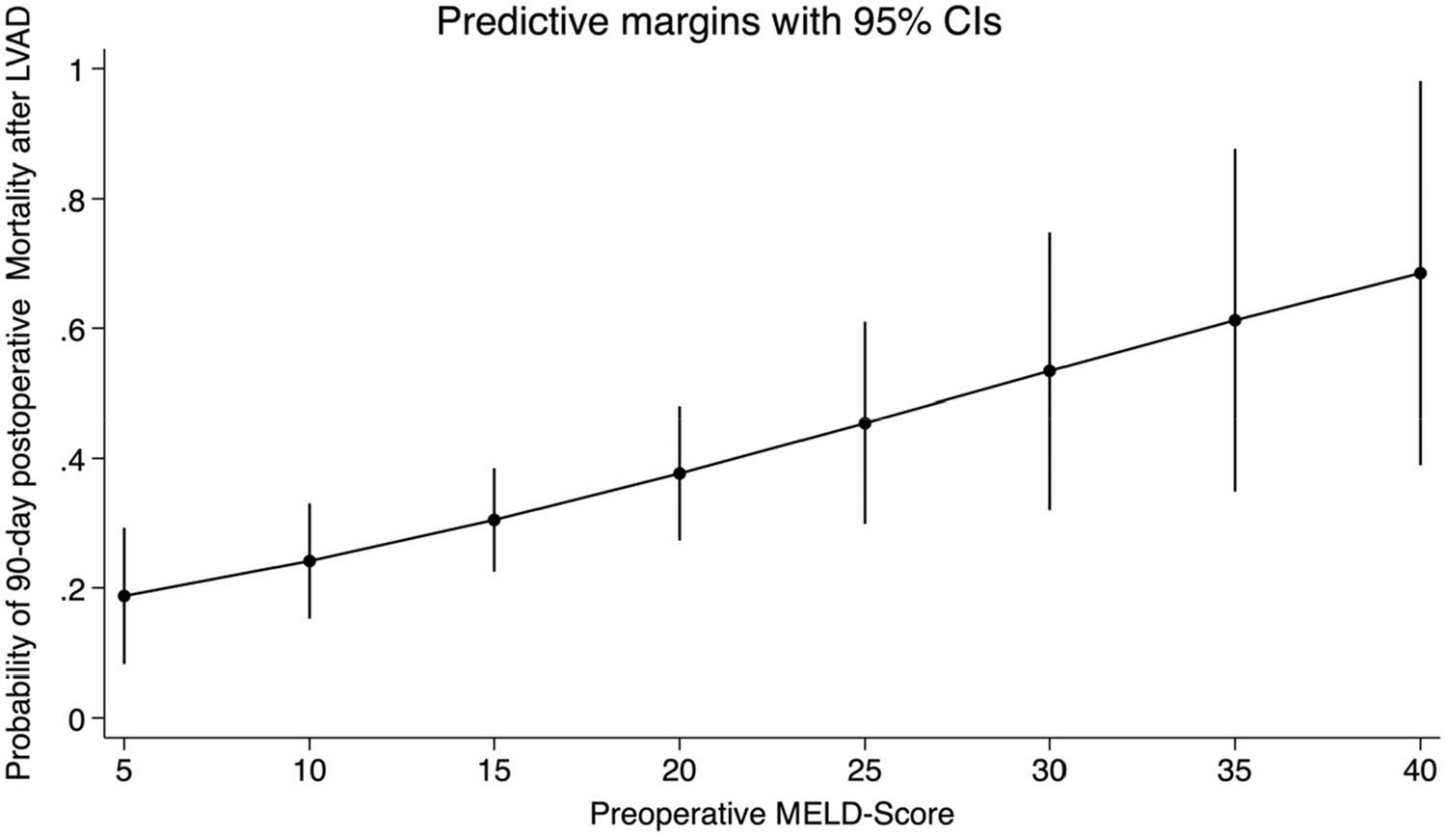
Prediction of mortality with respect to the MELD score‐marginal plot demonstrating the relation between the preoperative MELD score and the probability of the 90‐day mortality postoperatively after left ventricle assist device implantation, including the 95% confidence interval.

A multivariable logistic regression module included the MELD score, age of the patient at the LVAD implantation, EuroSCORE II, New York Heart Association Functional classification, and the etiology of myopathy, showed the MELD score as an independent predictor of 90‐day mortality (OR 1.06, 95% CI [1.0–1.1], *p* value 0.02), which can be interpreted as every one point increases in MELD score increases the probability of 90‐day mortality by 2%, a marginal plot developed to aid visualizing the relationship between the two variables with 95% confidence interval (Figure 4).

It is important to note that our study excluded patients who underwent concomitant cardiac procedures, including, but not limited to, tricuspid valve repair or replacement, during LVAD implantation. Therefore, our analysis does not provide insight into the impact of concomitant intervention on the incidence of postoperative RSHF. The role and optimal timing of valve surgery in patients undergoing LVAD implantation, particularly in those with pre‐existing valvular pathologies and RV dysfunction, remain an area of active clinical investigation and were beyond the scope of this study.

## 4 Discussion

Patients suffering from terminal heart failure, especially those requiring LVAD, may present with disruption of liver and renal function as a result of low cardiac output, which complicates the operative therapy. The presence of preoperative RHF further complicates the presentation and treatment in these patients. Using an established model to assess the risk in these patients may help to better guide our therapy choices and improve outcomes [5].

The observed variability in reported RHF incidence post‐LVAD highlights the historical lack of a universally adopted definition. Our study utilized a definition of severe RSHF based on the requirement for mechanical right ventricular support, focusing on events within the crucial early postoperative period (< 30 days). While recent efforts, such as those by the Mechanical Circulatory Support–Academic Research Consortium, have provided a more nuanced classification distinguishing early and late RHF, our study's scope was limited to the early, severe form necessitating mechanical intervention.

The human heart is the first functional organ to develop in all vertebrate embryos, becoming evident by Week 4 of development. Using RNA sequencing molecular technologies, researchers were able to identify two distinct mesoderm‐derived cardiac progenitors that eventually became the first and second cardiac fields. The descendants of the first cardiac field cells are found in the left ventricle, while the descendants of the second heart field give rise to the cells in the RV. This distinction in the embryonic origin between the two ventricles can facilitate an appreciation of the physiological differences, as well as the differences in cell shortening and relaxation in both ventricles [6].

The RV has been designated the “forgotten ventricle” due to the extensive research conducted on the mechanisms of left ventricle dysfunction, which has led to the development of several pharmaceuticals to enhance the survival of patients with LV failure. Conversely, the therapeutic options for a failing RV are limited. It is important to note that medications targeting beta‐blockers and those targeting the renin–angiotensin–aldosterone system (RAAS), which are the cornerstones of treating LV dysfunction, may be contraindicated in RV dysfunction [7].

Despite this understanding, the mechanisms of RV failure remain unclear. Currently, there are no approved therapies specifically targeting the RV. One reason for the lack of RV‐directed therapies is the complexity of the pathogenesis of RV failure, as observed in animal models and clinical studies. Thus, the mainstay of treatment for patients who develop RSHF after LVAD Implantation, beyond the well‐established medical treatment using, among others, diuretics, vasopressors, inotropic agents, and so forth, is mechanical circulatory support for the RV [8].

This was evidenced by many studies, including the ENDURANCE Trial, which compared centrifugal and coaxial devices in 2018. Despite the development of RHF being higher in the population receiving the centrifugal device was higher (38.5% vs. 26.4% *p* = 0.02), the incidence of RVAD implantation was similar in both groups (2.7% vs. 3.4% *p* = 0.77) [9].

The Interagency Registry for Mechanically Assisted Circulatory Support (INTERMACS) has defined RHF post‐LVAD as elevated central venous pressure and resultant end‐organ manifestation. This is the leading cause of mortality in patients who undergo implantation of an LVAD, particularly in cases where a centrifugal type of device was used, as is the case in the study population [10].

Several score systems have been developed to evaluate the occurrence of RSHF after the implantation of LVAD. These scores, however, lack one or more components of the MELD score. The EUROMACS‐RHF risk score is comprised of five components: severe RV dysfunction (2 points), a ratio of RA/PCWP ≥ 0.54 (2 points), advanced INTERMACS Class 1 through 3 (2 points), the need for ≥ 3 intravenous inotropes (2.5 points), and hemoglobin ≤ 10 g/dL (1 point) [11].

A comparative single‐centre study retrospectively examined the predictive capabilities of various available scores for RHF. This study compared the Michigan RVF risk score, Utah RVF risk score, Pittsburgh Decision Tree and the EUROMACS‐RHF risk score. The Michigan RVF score (*C*‐statistic 0.7374) compared favorably with newer RVF risk scores—Utah (0.5666), Pitt (0.5170), and Euromacs (0.6489). Pulmonary artery pulsatility index and preoperative RV dysfunction were then selected as the best performing hemodynamic and echocardiographic findings [12].

Considering the fact that the Michigan RVF risk score is the only score that takes into account both bilirubin and creatinine, it is unsurprising to see that it also performs better than the other scores which do not take both these parameters simultaneously into account [13].

The Pittsburgh Decision Tree is the only score that factors INR while assessing the risk for development of RSHF.

Taking this into account, it is worth noting that LVAD recipients with temporary RVAD were more likely to have postoperative bleeding requiring reoperation and acute renal failure. Mortality at 6 months was higher for LVAD patients with temporary RVAD than for those without RVAD: 53% in the LVAD patients with temporary RVAD versus 25% in patients with LVAD only (*p* < 0.001) [14].

Sodium is another parameter which is present in the MELD Na score that has not been considered in other risk assessment scores.

One possible approach to risk assessment and the development of RHF after implantation of LVAD is the STOP‐RVF Score. The STOP‐RVF risk calculator exhibited a significantly better performance than commonly used risk scores proposed by Kormos et al. (*C‐*statistic, 0.58; 95% CI, 0.53–0.63) and Drakos et al. (*C*‐statistic, 0.62; 95% CI, 0.57–0.67). This score, developed using machine learning received a *C*‐statistic of 0.75 (95% CI, 0.71–0.79) in the derivation cohort and 0.73 (95% CI, 0.67–0.80) in the validation cohort [15].

Patients with advanced heart failure often experience pulmonary hypertension (venous or mixed pattern) and some degree of RV dysfunction before LVAD implantation. The stress of LVAD surgery can easily disrupt the delicate coupling between RV and pulmonary vasculature afterload. Additionally, increased systemic venous return and/or excessive leftward shift of the interventricular septum can cause RV decompensation and failure [16]. Several retrospective trials have shown inhaled iloprost and milrinone to be promising [17, 18].

Further medical therapy depends on the differences in the regulation of energy production, mitochondrial function, reactive oxygen species (ROS) production, antioxidant protection, and angiogenesis between the LV and RV. These differences provide potential targets for the treatment of RHF. Various medications, such as Trimetazidine [19], Ranolazine [20], Perhexiline [21], and Elamipretide [22], have targeted one or more of these pathways to treat RSHF and shown mixed results in animal and human models. The best approach to treating RSHF is to prevent its onset.

Often in the clinical setting, it is not feasible to treat patients with heart failure for a long period of time before LVAD implantation. When possible, however, there is a quantifiable method to assess which patients will have a worse outcome. The MELD score can help us assess the initial risk and track the progress of patients depending on the preoperative preparation of the patients.

In one study, poor survival was observed in patients who did not show an improvement in MELD score since admission “Non‐Survivor” (median 30 [22–32]) versus “Survivor” (Median 11 [10–13]), *p* value 0.0029. Mortality was 100% in patients with MELD > 30, 66.67% with MELD 20–29, 50% with MELD 15–19, and 0% in group with MELD ≤ 14 [23].

Given the inevitable involvement of the liver in RSHF, it is important to examine the relationship between the development of hepatic failure and right‐sided heart disease.

The liver has a dual blood supply through the hepatic artery and the portal vein, which makes it resilient to ischemic damage [24].

In contrast, the liver's ability to resist pressure changes depends almost solely on its connected sinusoidal network, as the hepatic veins lack valves.

Congestion in the liver can cause damage through several mechanisms, including shear stress, decreased nitric oxide production from endothelial cells, reduced blood flow, impaired oxygen and nutrient diffusion, and sinusoidal stasis and congestion that promote thrombosis. Liver fibrosis is caused by mechanisms that lead to parenchymal extinction and activate hepatic stellate cells through protease‐activated receptors. The biochemical response of the liver to fibrosis exacerbates the pre‐ and afterload experienced by patients with cardiac failure [24].

The hepatic artery buffer response can compensate for up to a 60% decrease in portal flow. This response is triggered by a local increase in concentration of the vasodilator adenosine. In contrast, the portal vein cannot autoregulate its flow and relies on cardiac output and the gradient between portal and hepatic venous pressure. The sinusoids’ high permeability allows for oxygen extraction levels to reach nearly 90%, serving as a secondary defense mechanism against hypoxia [25].

Multiple factors inherent in LVAD implantation may contribute to RV dysfunction, including the loss of septal twist movement caused by acute and continuous unloading of the left ventricle, the loss of pericardial contribution to RV longitudinal contraction, the persistent strain of elevated right‐side pressure, and the potential for abnormal coronary perfusion to the struggling ventricle [26].

Future research employing these updated, comprehensive definitions will be valuable to fully characterize the incidence and predictors of RHF across the entire post‐LVAD timeline.

While our study included preoperative left ventricle ejection fraction as a characteristic, a detailed comparative analysis of comprehensive preoperative echocardiography and right heart catheterization parameters between patients who developed RSHF and those who did not was not systematically performed. We acknowledge that such data, including measures of right ventricular size, function, and pulmonary hemodynamics, are critical for a complete assessment of RHF risk and are currently a focus of future prospective studies at our center.

A recent external validation of the EUROMACS RSHF score on 662 patients with continuous flow LVADs revealed that its ability to predict RSHF is not particularly discriminatory, which invokes into doubt the clinical utility of the score as it provides an area under the receiver operating characteristic (ROC) curve for the RHF prediction of 0.64 (95% CI, 0.60–0.68) [27]

According to the current consensus of the ISHLT, a MELD score > 17 is the inflection point for increased risk of poor outcomes following mechanical circulatory support. Patients who have confirmed cirrhosis or end‐stage liver disease are poor candidates for LVAD except in very rare circumstances. Even in the absence of hepatic failure, patients with hepatic dysfunction before LVAD implantation are at increased risk of RSHF [28]. These conclusions were proven in the analysis of our data.

The findings of this study underscore the critical importance of the preoperative period for risk stratification and optimization in patients undergoing LVAD implantation. The MELD score's predictive value for early severe RSHF suggests that pre‐existing hepatic and renal dysfunction significantly impacts postoperative right heart function. Consequently, aggressive optimization of end‐organ function, where possible, should be a key component of preoperative preparation. While not a standardized approach in our cohort's timeframe, the observation in a patient with a high MELD score who received preoperative IABP support and did not develop RHF hints at the potential utility of strategies aimed at improving hemodynamics and reducing right ventricular afterload in high‐risk individuals.

The report further outlines the identifiable risk factors associated with a higher incidence of early severe postoperative RHF, which requires biventricular support after LVAD implantation, including chemotherapy‐induced cardiomyopathy, cardiogenic shock, high INTERMACS profile, and temporary cirrhosis. Circulatory support, in the form of V‐A ECMO, should be considered before durable LVAD in patients with pre‐existing therapies.

However, to date, no association has been shown between an increased MELD score preoperatively and the development of postoperative RSHF requiring RVAD implantation or indeed the postoperative outcomes of LVAD implantation.

## 5 Conclusions

The MELD score is a reliable preoperative predictor of mortality in patients receiving LVAD implantation, and it is particularly useful in screening patients who are likely to develop postoperative RSHF requiring RVAD implantation.

## Author Contributions

**Mohamed Elbayomi:** writing – review and editing, writing – original draft, conceptualization, methodology, data curation, formal analysis, investigation, software. **Presheet Pathare:** writing – review and editing, writing – original draft, conceptualization, methodology, data curation, investigation, formal analysis. **Raphael Groß:** investigation, writing – original draft, writing – review and editing, data curation, validation. **Friedrich Mellert:** project administration, supervision, validation. **Oliver Dewald:** supervision, resources, funding acquisition.

## Ethics Statement

This retrospective study complied with the Helsinki Declaration (2000), and the local Ethics Committee approved to perform this analysis (EC, No. 22‐448‐Br) based on retrospective data retrieval; the EC waived the need for written patients' informed consent.

## Conflicts of Interest

The authors declare no conflicts of interest.

## Transparency Statement

The lead author Presheet Pathare affirms that this manuscript is an honest, accurate, and transparent account of the study being reported; that no important aspects of the study have been omitted; and that any discrepancies from the study as planned (and, if relevant, registered) have been explained.

## Data Availability

The data that support the findings of this study are available from the corresponding author upon reasonable request.
